# Cholesterol accumulation caused by low density lipoprotein receptor deficiency or a cholesterol-rich diet results in ectopic bone formation during experimental osteoarthritis

**DOI:** 10.1186/ar4367

**Published:** 2013-11-04

**Authors:** Wouter de Munter, Arjen B Blom, Monique M Helsen, Birgitte Walgreen, Peter M van der Kraan, Leo AB Joosten, Wim B van den Berg, Peter LEM van Lent

**Affiliations:** 1 Department of Rheumatology Research and Advanced Therapeutics, Radboud University Medical Center, Nijmegen, the Netherlands; 2 Department of Medicine and Nijmegen Institute for Infection, Inflammation and Immunity, Radboud University Medical Center, Nijmegen, the Netherlands; 3 Nijmegen Institute for Infection, Inflammation and Immunity, Radboud University Medical Center, Geert Grooteplein 26-28, PO Box 9101, 6500 HB Nijmegen, the Netherlands

## Abstract

**Introduction:**

Osteoarthritis (OA) is associated with the metabolic syndrome, however the underlying mechanisms remain unclear. We investigated whether low density lipoprotein (LDL) accumulation leads to increased LDL uptake by synovial macrophages and affects synovial activation, cartilage destruction and enthesophyte/osteophyte formation during experimental OA in mice.

**Methods:**

LDL receptor deficient (LDLr^−/−^) mice and wild type (WT) controls received a cholesterol-rich or control diet for 120 days. Experimental OA was induced by intra-articular injection of collagenase twelve weeks after start of the diet. OA knee joints and synovial wash-outs were analyzed for OA-related changes. Murine bone marrow derived macrophages were stimulated with oxidized LDL (oxLDL), whereupon growth factor presence and gene expression were analyzed.

**Results:**

A cholesterol-rich diet increased apolipoprotein B (ApoB) accumulation in synovial macrophages. Although increased LDL levels did not enhance thickening of the synovial lining, S100A8 expression within macrophages was increased in WT mice after receiving a cholesterol-rich diet, reflecting an elevated activation status. Both a cholesterol-rich diet and LDLr deficiency had no effect on cartilage damage; in contrast, ectopic bone formation was increased within joint ligaments (fold increase 6.7 and 6.1, respectively). Moreover, increased osteophyte size was found at the margins of the tibial plateau (4.4 fold increase after a cholesterol-rich diet and 5.3 fold increase in LDLr^−/−^ mice). Synovial wash-outs of LDLr^−/−^ mice and supernatants of macrophages stimulated with oxLDL led to increased transforming growth factor-beta (TGF-β) signaling compared to controls.

**Conclusions:**

LDL accumulation within synovial lining cells leads to increased activation of synovium and osteophyte formation in experimental OA. OxLDL uptake by macrophages activates growth factors of the TGF-superfamily.

## Introduction

Osteoarthritis (OA) is a common disease of unknown etiology. The association of OA with metabolic syndrome has long been established but the exact mechanism remains unclear [1,2]. The idea that obesity enhances OA development solely due to increased loading [3] is obsolete and more often studies show the association between obesity and OA development in non-weight-bearing joints [4-7].

Decreased levels of high-density lipoprotein (HDL) and increased levels of low-density lipoprotein (LDL) particles are, amongst other features, part of the metabolic syndrome [8]. In a comparative analysis of serological parameters, several studies demonstrated that OA patients have significantly higher serum levels of LDL compared to healthy controls [9,10]. Studies focusing on cardiovascular diseases, such as atherosclerosis, show pro-inflammatory capacities of LDL and modified LDL [11,12]. LDL particles form the main transport vehicle of cholesterol from the liver to the tissues. LDL can be oxidized in an inflammatory milieu and, therefore, high levels of LDL result in enhanced oxidized LDL (oxLDL) levels in pathological conditions where free radicals are present [13,14]. OxLDL is taken up by macrophages via scavenger receptor class A, B (CD36) and E (lectin-like oxLDL receptor-1; LOX-1), resulting in a phenotype shift into a more inflammatory cell type [15-19].

A substantial population of OA patients develops a thickened lining layer comprising macrophages that exhibit an activated phenotype. Macrophages derived from biopsies with early OA produce elevated amounts of pro-inflammatory mediators [20]. Depletion of macrophages from OA synovium using anti-CD14–conjugated magnetic beads led to decreased levels of TNF-α, IL-1β, IL-6 and IL-8 [21]. In previous studies we have shown that synovial macrophages are crucial in the development of joint pathology in experimental OA. Selective depletion of lining macrophages using the clodronate-suicide technique prior to induction of collagenase-induced OA strongly inhibited development of cartilage destruction and osteophyte formation, probably regulated by a strong decrease in metalloproteinase (MMP)-3 and −9 expression [22].

Transforming growth factor-β (TGF-β) and bone morphogenetic proteins (BMP) are important growth factors involved in the formation of new cartilage or bone in ligaments (enthesophyte formation) or along the bone surface (ectopic bone formation or osteophyte formation) [23]. In previous studies we showed that multiple injections of members of the TGF-super family, such as TGF-β or BMP-2, directly into the knee joint of the mouse caused abundant enthesophyte/osteophyte formation [24,25]. Moreover, we postulated that local depletion of synovial macrophages prior to injections of these growth factors significantly inhibited new formation of cartilage/bone, suggesting that macrophage factors highly contribute to this process [26,27].

The presence of high levels of LDL in OA joints with an enhanced inflammatory environment may lead to uptake of oxLDL by synovial lining macrophages, thereby contributing to development of OA pathology. LDL receptor deficient (LDLr^−/−^) mice, which are generally used in atherosclerotic research [28], are unable to clear and metabolize cholesterol-rich intermediate and low density lipoproteins, causing hypercholesterolemia that can be enhanced by a cholesterol-rich diet [29]. In this study, we investigated the effect of increased serum LDL levels on OA development in experimental collagenase-induced OA. We focused on synovial thickening/activation, cartilage damage and enthesophyte/osteophyte formation in both LDLr^−/−^ mice and mice receiving a cholesterol-rich diet.

## Methods

### Animals

Female mice homozygous for the *Ldlr*^*tm1Her*^ mutation (LDLr^−/−^) and their wild type (WT) control C57BL/6 J were obtained from The Jackson Laboratory (Bar Harbor, ME, USA). Mice were 10 to 13 weeks old when used in the experiments, were housed in filter-top cages and received food and water *ad libitum*. Animal studies were approved by the Institutional Review Board (Animal Experiment Committee Radboud University Medical Center) and were performed according to the related codes of practice.

### Collagenase-induced osteoarthritis

Mice were fed a standard diet or a cholesterol-rich diet (+0.15% cholesterol; AB Diets, Woerden, the Netherlands). Instability OA was induced by intra-articular injection of 1 unit bacterial collagenase (Sigma-Aldrich, St. Louis, MO, USA) into the right knee on days 84 and 86 after start of the diet [30]. On day 120, mice were weighed and sacrificed, after which both left and right knee joints were isolated and processed for histological analysis. Serum samples were obtained to determine cholesterol levels.

### Histological and immunohistochemical preparation

Isolated knee joints were fixed in 4% buffered formalin and subsequently decalcified in formic acid and embedded in paraffin. Eight representative sections (7 μm) of each joint from various depths were stained with H & E or Safranin O-fast green for histological analysis. For immunohistochemistry, sections were deparaffinized and endogenous peroxidase activity was blocked using 1% H_2_O_2_ in methanol. Next, sections were incubated in buffered citrate (pH 6.0) for antigen retrieval. Incubation with 0.1% Triton X-100 was used for cell membrane permeabilization, followed by incubation with rabbit anti-mouse apolipoprotein B (ApoB; Abcam, Cambridge, UK), rabbit anti-mouse S100A8 (kindly provided by Dr. Vogl, Institute of Immunology, University of Muenster, Germany), or normal rabbit immunoglobulin G (IgG) (R&D Systems, Minneapolis, MN, USA) as control. Subsequently, sections were incubated with the secondary antibody, biotinylated goat anti-rabbit IgG, and binding was detected using the ABC-HRP kit (Elite kit; Vector Laboratories, Burlingame, CA, USA). Peroxidase was developed with diaminobenzidine (DAB; Sigma-Aldrich) and sections were counter-stained with hematoxylin. Sections were randomly coded and scored in a blinded way by two independent investigators.

### Histological analysis

Synovial thickening and immunohistochemical staining were measured using an arbitrary scoring system (0 to 3, where 0 = no thickening/staining and 3 = most observed thickening/staining). Three sections of various depths were scored per knee joint.

Cartilage damage in the tibial-femoral joint was scored using the OA score developed by Pritzker *et al.*[31], adapted by us for mice (from 0 = no damage, to 30 = maximal damage) [32]. Five sections were scored per knee joint.

Size of ectopic bone formation was measured by the use of image analysis (Leica Qwin, Leica Microsystems, Rijswijk, The Netherlands). Osteophytes and enthesophytes were manually circled by an investigator blinded for the experimental condition, after which the surface areas were calculated by Leica-software. The mean cross-sectional surface area in three sections per knee joint was determined, including knee joints without ectopic bone formation.

### Synovial wash-outs

Synovium was isolated from murine knee joints 7 and 36 days after collagenase injection. Tissue was weighed and put in Roswell Park Memorial Institute medium (RPMI; Gibco, Invitrogen, Carlsbad, CA, USA), enriched with 48 μg/mL gentamicin (Centrafarm, Etten-Leur, The Netherlands) and 0.1% BSA (Sigma-Aldrich) for one hour at room temperature.

### Bone marrow derived macrophages

Macrophages were differentiated out of bone marrow cells using 15 ng/mL recombinant murine macrophage colony stimulating factor (M-CSF; R&D Systems). For this, tibiae from WT or LDLr^−/−^ mice were removed and bone marrow cells were flushed with (D)MEM; Gibco) supplemented with 10% FCS (Thermo Scientific, Waltham, MA, USA), 1 mM pyruvate (Gibco) and 48 μg/mL gentamicin.

### Oxidized LDL preparation and macrophage stimulation

OxLDL was prepared from a large batch of LDL which was first isolated by single-spin density-gradient ultracentrifugation from ethylenediaminetetraacetic acid (EDTA)-treated blood donated by healthy volunteers and frozen in 10 mM phosphate buffer (pH 7.4) containing 0.9% NaCl, 10% (w/v) saccharose and 0.1 mM EDTA. LDL was thawed and dialyzed for seven hours against saline in a Slide-A-Lyzer cassette (Pierce Chemical Company, Rockford, IL, USA). Subsequently, LDL was oxidized with 0.5 mg/mL copper sulfate (Merck, Darmstadt, Germany) for 24 hours at room temperature, after which oxLDL was dialyzed again for one hour [33]. OxLDL concentration was determined by the use of a bicinchoninic acid assay (Thermo Scientific) and a Sunrise microplate reader (Tecan Group, Männedorf, Switzerland). OxLDL was pre-incubated for 10 minutes with 10 μg/mL polymixine B sulfate (Sigma-Aldrich) before experimental use in order to rule out lipopolysaccharide (LPS) interference. Macrophages were stimulated with 50 μg/mL oxLDL, or an equal volume of saline (also pre-incubated with 10 μg/mL polymixine B sulfate) for 24 or 48 hours at 37°C. Stimulations were performed in 5% non heat-inactivated FCS. Supernatant was used for functional TGF-β and BMP analyses and cells were lysed in 500 μL TRI-reagent (Sigma-Aldrich) for quantitative detection of messenger RNA levels. All reactions were performed in quadruple.

### Quantitative real-time polymerase chain reaction

RNA was extracted using the single step RNA isolation method described by Chomczynski and Sacchi [34]. Isopropanol (Merck) was used for precipitation, after which the RNA pellet was washed twice with 75% ethanol. The pellet was reconstituted in RNAse free water and subsequently treated with DNase (Invitrogen). RNA was reversed transcribed into complementary DNA (cDNA) using reverse transcriptase, oligo(dT) primers and dNTPs (Invitrogen).

Quantitative real-time PCR (qPCR) was performed using StepOnePlus Real-Time PCR system and StepOne software v2.2 (Applied Biosystems, Foster City, CA, USA), under the following conditions: 10 minutes 95°C, followed by 40 cycles of 15 seconds 95°C and 1 minute 60°C. Data were collected during the last 30 seconds of each cycle. Product specificity was confirmed by assessment of dissociation-characteristics. The reaction was performed in a total volume of 10 μL, containing 3 μL diluted cDNA, 1 μL forward primer (5 μM), 1 μL reverse primer (5 μM) and 5 μL SYBR Green Master Mix (Applied Biosystems). The following primers were designed with Primer Express 2.0 (Applied Biosystems) and manufactured by Biolegio (Nijmegen, the Netherlands): GAPDH: 5′-GGCAAATTCAACGGCACA-3′ (forward) and 5′-GTTAGTGGGGTCTCGCTCCTG-3′ (reverse); IL-12p40: 5′-AGCTAACCATCTCCTGGTTTGC-3′ (forward) and 5′-CCACCTCTACAACATAAACGTCTTTC-3′ (reverse); TGF-β1: 5′-GCAGTGGCTGAACCAAGGA-′3 (forward) and 5′-AAGAGCAGTGAGCGCTGAATC-3′ (reverse); BMP2: 5′-CGCAGCTTCCATCAC-3′ (forward) and 5′-GCCGGGCCGTTTTCC-3′ (reverse); BMP4: 5′-CCGCTTCTGCAGGAACCA-3′ (forward) and 5′-AGTGCGTCGCTCCGAATG-3′ (reverse); BMP7: 5′-ACGGACAGGGCTTCTCCT-3′ (forward) and 5′-ATGGTGGTATCGAGGGTG-3′ (reverse). Relative expression levels were presented as ΔΔCt, being the threshold cycle (Ct) value corrected for GAPDH and unstimulated control.

### CAGA-Luc and BRE-Luc reporter constructs

3T3 cells (ATCC, Manassas, VA, USA) were transduced with a CAGA-Luciferase reporter plasmid (CAGA-Luc) or a BMP responsive-luciferace reporter plasmid (BRE-Luc) for two and a half hours with a multiplicity of infection of 10. Both plasmids were kindly provided by Dr. Ten Dijke (Department of Molecular Cell Biology, Leiden University Medical Center, the Netherlands). After 24 hours, the transduction efficiency of CAGA-Luc was checked with fluorescence microscopy, confirming the presence of GFP that is constitutively active in this construct. The transduced cells were then stimulated overnight with macrophage supernatant or synovial wash-outs under serum-free conditions, after which luminescence was measured.

### Statistical analysis

Statistical differences between two values were calculated using a student’s *t*-test or Mann Whitney *U* test depending on Gaussian distribution. Statistical differences between more than two values were tested using a one-way analysis of variance (ANOVA) or Kruskal-Wallis test, followed by a Bonferoni or Dunns post test, respectively. All analyses were performed using Graph Pad Prism 5 (GraphPad Software, La Jolla, CA, USA) and *P*-values less than 0.05 were considered to be significant. Data are depicted as mean ± standard error of the mean (SEM).

## Results

### A cholesterol-rich diet increased serum cholesterol levels and enhanced ApoB uptake by LDLr deficient synovial lining cells

**Figure 1:**
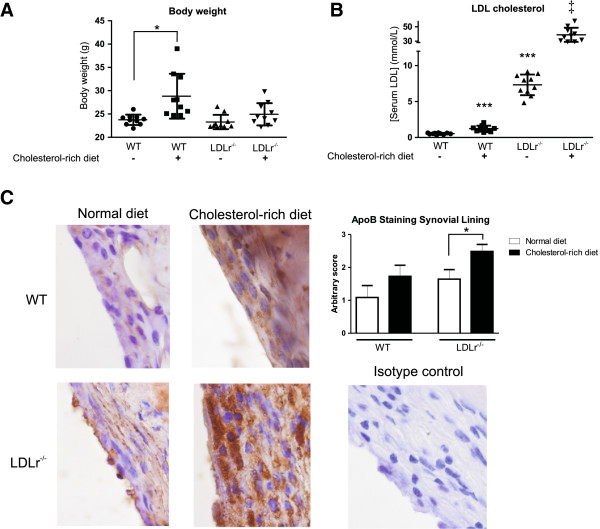
**A cholesterol-rich diet leads to increased serum LDL levels and ApoB accumulation in synovial lining.** Both WT and LDLr deficient mice received a normal or cholesterol-rich diet for 120 days. Increased body weight following a cholesterol-rich diet was observed in WT mice but not in LDLr deficient mice **(A)**. Serum LDL levels were increased both in WT and LDLr deficient animals after receiving a cholesterol-rich diet **(B)**. Synovial lining cells (predominantly macrophages) of LDLr deficient mice showed increased intracellular ApoB accumulation, suggesting increased oxLDL uptake **(C)**. *P<0.05; ***P<0.001 versus WT mice with normal diet; ‡*P*<0.001 versus LDLr deficient mice with normal diet and WT mice with cholesterol-rich diet. n = 10 mice per group. ApoB, apolipoprotein B; LDL, low density lipoprotein; LDLr, low density lipoprotein receptor; oxLDL, oxidized low density lipoprotein; WT, wild type.

To investigate the effects of enhanced serum cholesterol levels during pathogenesis of experimental collagenase-induced OA, both WT and LDLr^−/−^ mice were fed a standard diet or cholesterol-rich diet for 120 days. A cholesterol-rich diet resulted in a 21% increase of body weight in WT mice (from 23.8 g ± 0.4 to 28.8 g ± 1.5; *P* = 0.05), but, in contrast, did not significantly affect the weight of LDLr^−/−^ mice (Figure 1A). Serum analysis for cholesterol levels showed that a cholesterol-rich diet in WT mice increased serum LDL concentration by 128% (from 0.54 mmol/L ± 0.04 to 1.23 mmol/L ± 0.13; *P* = 0.006). In LDLr deficient mice, enhanced serum LDL concentration following a cholesterol-rich diet was even more pronounced (428% raise, from 7.33 mmol/L ± 0.46 to 38.73 mmol/L ± 3.11; *P*<0.0001; Figure 1B). Apart from enhanced serum LDL levels, local effects of a cholesterol-rich diet and LDLr deficiency were also investigated. Expression of ApoB, which is the main protein in oxLDL particles, was examined by immunohistochemistry. Both a cholesterol-rich diet and LDLr deficiency enhanced uptake of ApoB by the synovium. Particularly, cells in the lining layer accumulated ApoB within their cytoplasm. Mice deficient for the LDLr showed a significant increase in ApoB staining after receiving a cholesterol-rich diet, suggesting enhanced up-take of oxLDL (Figure 1C). These results suggest a positive relation between oxLDL accumulation by lining macrophages and serum LDL levels.

### High serum cholesterol levels did not alter synovial thickening but increased the activation status of the macrophage particularly in wild type mice during collagenase-induced osteoarthritis

**Figure 2:**
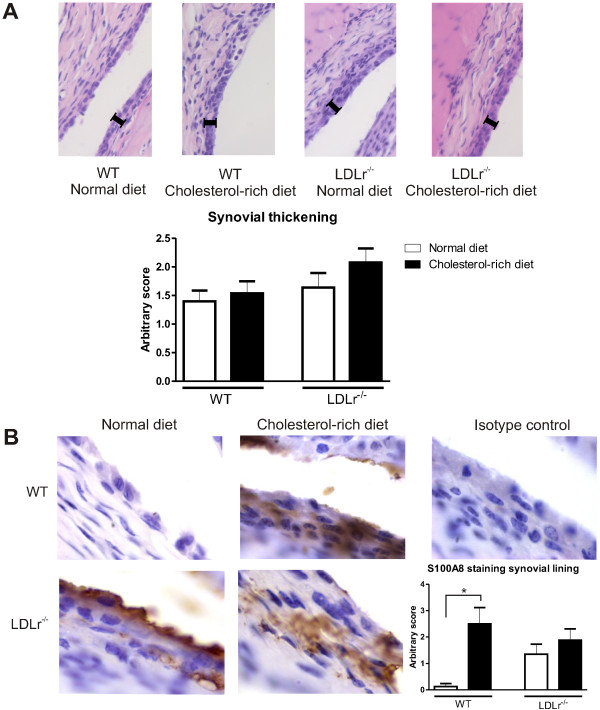
**Cholesterol-rich conditions lead to an activated synovium, rather than synovial thickening.** Thickening of the synovial lining layer was determined in hematoxylin and eosine stained sections, using an arbitrary score between 0 and 3. All mice showed moderate synovial thickening with no significant differences between various groups **(A)**. Immunohistochemistry, however, showed an increase in S100A8 expression in WT mice after receiving a cholesterol rich diet, suggesting enhanced macrophage activation **(B)**. *P<0.05. n = 10 mice per group.WT, wild type.

Since high serum levels of LDL resulted in enhanced uptake of (modified) LDL by the synovial lining layer cells, we further investigated whether enhanced serum LDL levels during collagenase-induced experimental OA affected synovial thickening. Histology of knee joints at day 36 after induction of experimental OA showed moderate synovial thickening in WT mice on a normal diet (1.4 ± 0.2; Figure 2A). Synovial thickness, however, was not significantly higher in mice that received a cholesterol-rich diet, nor was it significantly higher in mice deficient for the LDL receptor. To study the contribution of the macrophage to lining layer activation, immunostaining with S100A8 was performed. S100A8 is expressed only by activated macrophages but not by fibroblasts [35]. WT mice showed an increased S100A8 expression after receiving a cholesterol-rich diet (from 0.43 ± 0.16 to 1.13 ± 0.35; *P* = 0.035). In LDLr^−/−^ mice increased S100A8 expression after a cholesterol-rich diet did not reach significance Figure 2B).

### High serum cholesterol levels caused by a cholesterol-rich diet did not alter cartilage destruction in collagenase-induced osteoarthritis

**Figure 3:**
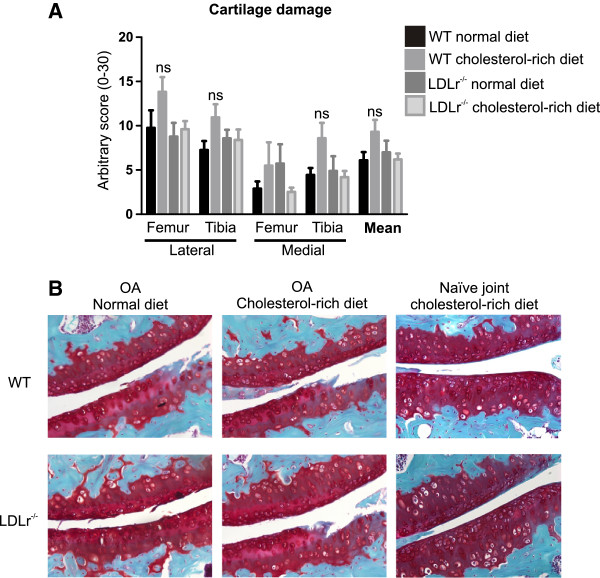
**Increased serum cholesterol levels do not affect cartilage damage during experimental OA.** Cartilage damage was scored at the medial and lateral side of the tibial plateau and femoral condyles **(A)**. No significant differences were found between the different groups. **B** shows representative pictures (stained with safranin O - fast green) of the medial femoral condyle and tibial plateau of four OA joints. Two representative pictures of naïve joints show that the observed cartilage damage was due to collagenase injection rather than age or genomic and environmental factors. ns = not significant. n = 10 mice per group. OA, osteoarthritis.

Since we show that a cholesterol-rich diet is capable of activating macrophages, we further investigated whether high serum cholesterol levels affect cartilage damage. Overall cartilage destruction was mild (mean score WT mice without cholesterol-rich diet 6.1 ± 1.5; Figure 3). Although WT mice that received a cholesterol-rich diet showed marginal differences in cartilage damage compared to mice receiving a normal diet at the lateral side of the femur and both lateral and medial side of the tibia, these values did not reach significance. In line with this, no differences in cartilage damage were seen in LDLr deficient mice between the group receiving the normal diet and the group receiving the cholesterol-rich diet. Moreover, no differences between cartilage damage in WT mice on the normal diet and LDLr deficient mice on the normal diet were observed (Figure 3).

### Increased serum cholesterol levels result in enhanced bone formation in ligaments during experimental osteoarthritis

**Figure 4:**
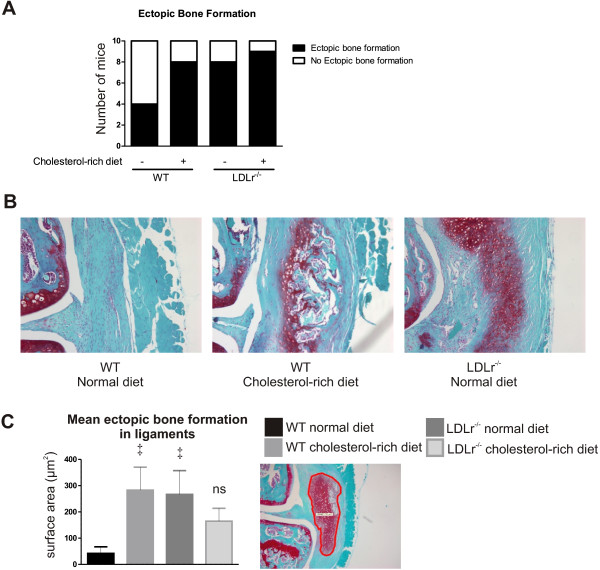
**Enhanced serum cholesterol levels increase ectopic bone formation in ligaments.** The size of cartilage and bone formations was measured using digital image analysis. The incidence of ectopic bone formation was increased after a cholesterol-rich diet, LDL receptor deficiency and the combination of these two **(A)**. The photographs depict representative examples of ectopic bone formation at the medial collateral ligament **(B)**. When cross-sectional surface areas were measured, a cholesterol-rich diet or LDL receptor deficiency showed increased total enthesophyte size in ligaments **(C)**. ns = not significant compared to other groups; ‡*P*<0.05 compared to WT mice with normal diet. LDL, low density lipoprotein; WT, wild type.

Earlier studies have shown that synovial macrophages are crucial in mediating new formation cartilage/bone in ligaments and along the bone surface. Since formation of enthesophytes are a consistent and early feature of OA [36], leading to ligament rigidity and less functionality of the joint, we first examined the effects of high cholesterol levels on ectopic bone formation in collateral and cruciate ligaments. While only four out of ten WT mice with OA on a normal diet showed enthesophyte formation, there were eight out of ten WT mice on a cholesterol-rich diet that presented enthesophyte formation. In LDLr deficient mice on a normal diet, eight out of ten mice showed enthesophyte formation, whereas there were nine out of ten mice in the LDLr deficient group on a cholesterol-rich diet that showed this condition (Figure 4A). Both a cholesterol-rich diet and LDLr deficiency resulted in a significantly increased size of enthesophytes (fold increase of 6.7 and 6.1, respectively; *P*<0.05). In contrast, the combination of LDLr^−/−^ and a cholesterol-rich diet only showed a trend towards an increased total size of enthesophytes compared to WT mice on a normal diet; however, values did not reach significance (Figure 4B and C).

### High serum cholesterol levels result in increased osteophyte formation and presence of active TGF-β in collagenase-induced osteoarthritis

**Figure 5:**
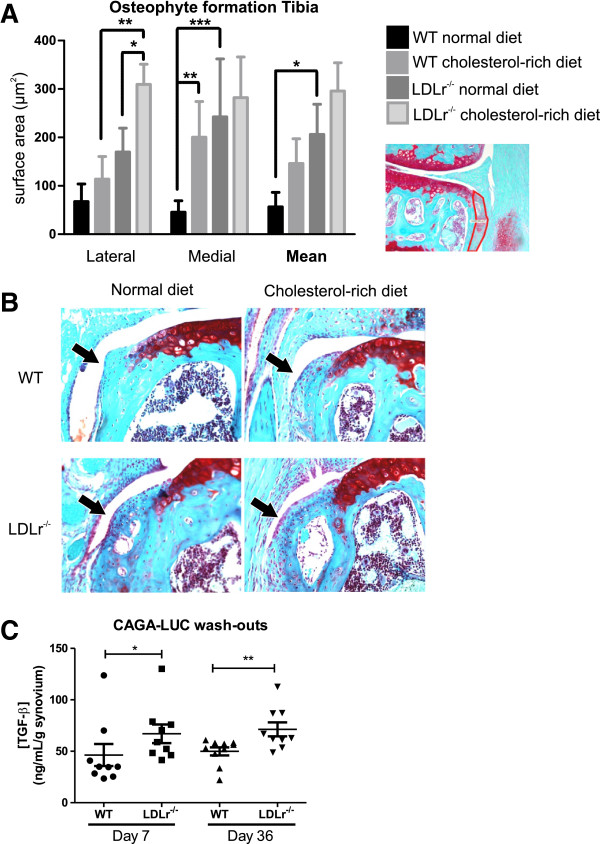
**Increased serum cholesterol levels enhance osteophyte formation at the margins of the tibial plateau and increase active TGF-β presence.** Osteophyte size was digitally measured at the margins of the tibial plateau **(A)**. **B** shows representative photos (stained with safranin O - fast green) of osteophytes at the lateral side of the tibial plateau. The presence of active TGF-β was found to be increased in LDLr^−/−^ mice on a cholesterol-rich diet compared to WT mice on a cholesterol-rich diet by testing synovial wash-outs on a CAGA-LUC assay **(C)**. *P<0.05; **P<0.01; ***P<0.001. n = 10 mice per group. LDLr, low density lipoprotein receptor; WT, wild type.

Since enthesophyte formation in joint ligaments strongly correlates with osteophyte formation [23], we next investigated whether serum cholesterol levels affected osteophyte formation. We measured osteophyte size at the margins of the lateral and medial side of the tibial plateau and femoral condyles using image analysis. LDLr deficient mice, receiving a cholesterol-rich diet, showed significant enhancement of osteophyte formation at the lateral side of the tibial plateau, when compared to WT mice with a cholesterol-rich diet (fold increase 2.7; *P*<0.01). Even more pronounced was the increase in osteophyte formation in LDLr deficient mice on a normal diet, compared to WT mice on the same diet (fold increase 5.3; *P*<0.001; Figure 5A and B). Besides increased osteophyte formation due to LDLr deficiency, a cholesterol-rich diet further increased osteophyte formation at the lateral site of the tibial plateau in LDLr deficient mice (fold increase 1.8; *P*<0.05). At the medial side of the tibia, a cholesterol-rich diet resulted in increased osteophyte formation in WT mice (fold increase 4.4; *P*<0.01). Thus, both a cholesterol-rich diet and LDLr deficiency cause increased osteophyte formation at the margins of the tibial plateau. At the margins of the femoral condyles, no significant differences in osteophyte formation were found between the various groups (data not shown). In previous studies we have shown that TGF-β and BMPs are major growth factors involved in driving new formation of cartilage/bone [24,25]. We therefore tested synovial wash-outs of WT and LDLr^−/−^ mice for the presence of active TGF-β using a CAGA-Luc assay (Figure 5C). Both on the 7th and 36th day after collagenase injection, levels of activated TGF-β were significantly higher in LDLr^−/−^ mice on a cholesterol-rich diet, compared to WT mice on a cholesterol-rich diet.

### OxLDL stimulated macrophages activate TGF-β *in vitro*

**Figure 6:**
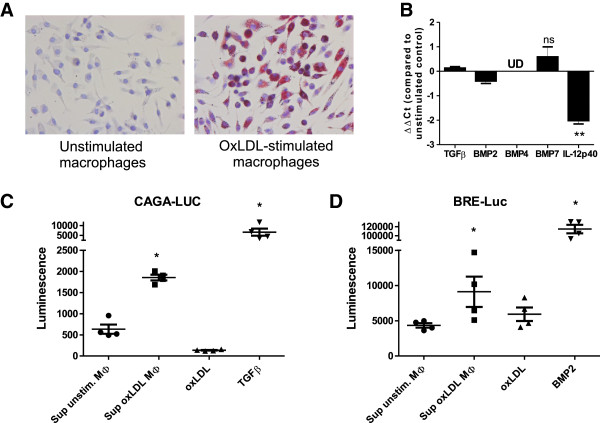
**LDL- and oxLDL-laden macrophages enhance anabolic processes by activating TGF-β and to a lesser extent BMP, rather than increase production of TGF-β or BMP.** Bone marrow derived cells were differentiated into macrophages, after which they were stimulated with 50 mg/mL oxLDL or an equal volume of PBS. Staining the cells with oil red O showed uptake of oxLDL **(A)**. RNA expression did not show significant upregulation of TGF-β, BMP2, BMP4 or BMP7 **(B)**; however, a functional TGF-β reporter gene luciferase (CAGA-Luc) assay did show increased presence of active TGF-β **(C)**. Furthermore, the BMP responsive luciferase (BRE-Luc) assay also showed increased activity in supernatant of oxLDL stimulated macrophages, albeit to a lesser extent **(D)**. UD = undetectable; *P<0.05 versus unstimulated control; **P<0.01; ns = no significant regulation. BMP, bone morphogenetic protein; LDL, low density lipoprotein; oxLDL, oxidized low density lipoprotein.

As ApoB accumulation in synovial macrophages correlated with increased ectopic bone formation in both WT and LDLr^−/−^ mice, we investigated whether oxLDL stimulated macrophages are capable of producing anabolic factors that may be responsible for this process. For that reason, bone marrow derived macrophages were cultured with oxLDL for 24 hours. Staining with oil red O indeed showed a high uptake of oxLDL by macrophages, which did not alter cell viability (Figure 6A). Analysis of RNA expression did not show upregulation of important growth factors known to be involved in ectopic bone formation such as TGF-β, BMP2, -4 and −7, while IL12p40, a positive control for murine oxLDL-laden macrophages [37], did show a significant down regulation with a ΔΔCt of 2.0 ± 0.13 (Figure 6B). Next, we studied the presence of active TGF-β and BMP using specific bioassays. When supernatant of macrophages that were stimulated with oxLDL for 48 hours was functionally tested for TGF-β activity using the CAGA-Luc assay, we found a significant 2.9 fold increase in signal (*P*<0.05), while oxLDL alone did not increase TGF-β activity (Figure 6C). The BRE-Luc assay, which measures active BMP, did also show an increase in signal by supernatant of oxLDL-laden macrophages, albeit to a lesser extent (fold increase 1.8; *P*<0.05; Figure 6D).

## Discussion

In the present study, we show that enhanced serum LDL cholesterol levels increase ectopic bone formation in mice with collagenase-induced OA. By using both a cholesterol-rich diet and LDLr deficiency, we demonstrated that an enhanced LDL cholesterol level in particular, rather than LDLr deficiency or a specific food element, is responsible for the enhancement of ectopic bone formation.

From previous studies we know that synovial lining cells are important for OA pathology. Selective elimination of synovial macrophages from the intimal lining of the knee joint prior to induction of collagenase-induced OA ameliorated the development of both cartilage damage and ectopic bone formation. In mice that lack the LDLr, a cholesterol-rich diet led to a strong increase in ApoB staining within the lining layer. Every LDL particle contains a single ApoB molecule that can be used as a marker for LDL and oxLDL uptake [38]. The accumulation of ApoB in synovial lining cells of LDLr deficient mice suggests that lining cells in an OA joint take up oxLDL in a cholesterol-rich environment. Macrophages can phagocytize LDL via the LDLr which is suppressed when LDL serum levels rise, leading to regulation of LDL uptake into the cell [39]. Under inflammatory conditions, LDL is oxidized and phagocytized by scavenger type A and B receptors lacking this negative feedback loop [40]. A macrophage is a very plastic cell type which can differentiate into activated M1 or suppressive M2 subtypes. Recently we found in *in vitro* studies that M2 macrophages express higher levels of SR-A and CD36 receptors compared to M1 macrophages and that enhanced uptake of oxLDL by M2 macrophages changed the suppressive character of this cell type into an M1-like macrophage releasing enhanced levels of IL-1 and IL-6 [16]. Although uptake of oxLDL in the lining layer of the LDLr^−/−^ mice receiving a cholesterol-rich diet was strongly enhanced, no elevation of S100A8 staining was observed. S100A8 is a member of the alarmin family which is particularly produced by activated macrophages but not by non-activated macrophages and has been described as a marker for macrophage activation [35]. The lack of S100A8 staining in LDLr^−/−^ mice suggests that uptake of large amounts of oxLDL by lining macrophages does not enhance the production of pro-inflammatory molecules and chemokines and, therefore, does not alter synovial thickening and cartilage destruction. In contrast, high cholesterol levels in WT mice induced only minor uptake of oxLDL by lining macrophages, but strongly enhanced S100A8 levels. This accumulation of S100A8 within the lining layer might explain the trend of increased cartilage destruction observed in WT mice on a cholesterol-rich diet. S100A8 production by synovial macrophages may be driven by moderate uptake of oxLDL or LDL, while excessive uptake of oxLDL may inhibit production of pro-inflammatory factors by synovial macrophages. Another explanation of the slight increase in cartilage damage observed in WT mice on a cholesterol-rich diet (Figure 3) is that it might also be due to the increased body weight (Figure 1A). Nevertheless, by using a cholesterol-rich diet rather than a high-fat diet, we minimized weight gain, preventing amelioration of the OA process via increased loading [41]. Lack of cartilage damage in cholesterol-rich conditions may also be explained by the increased presence of active TGF-β which protects the cartilage from proteoglycan depletion and matrix breakdown, as described in an earlier study by Van Beuningen *et al*. [42]. From our own experience, we know that the experimental model which is used here is less severe in female mice compared to males. Although all mice in this experiment developed cartilage lesions, the OA scores were relatively low. It would be interesting to repeat our experiment in a more severe model, investigating whether increased cholesterol-associated cartilage damage is detected.

Apart from cartilage destruction, new formation of cartilage and bone is also observed in ligaments and locations along the bone surface during collagenase-induced OA. Focusing on ligaments (enthesophyte formation), we noticed that an increase in LDL levels by a cholesterol-rich diet markedly enhanced new cartilage and bone formation. OxLDL might directly induce proliferation and differentiation of stem cells present within the ligaments, or indirectly by enhancing growth factor production in synovium. Several studies have shown that oxLDL has a toxic effect on proliferation and differentiation of stem cells, and, in line with that, our own findings show that oxLDL (50 μg/mL) is highly toxic for stem cells (data not shown), whereas similar concentrations do not affect macrophage viability (Figure 6A) [43-47]. For that reason, we assume that enthesophyte formation may be more related to the effects of oxLDL on synovial lining activity. In previous studies in our lab it was found that intra-articular injection of the growth factors TGF-β and BMP2 resulted in formation of osteophytes [24,25]. Moreover, in the absence of synovial macrophages, injection of TGF-β was less able to induce new formation of cartilage and bone, suggesting that macrophages are crucial in mediating TGF-β effects [26]. Furthermore, we found that spheroid formation in a mesenchymal cell line was increased when these cells were co-cultured with macrophages, whereas total TGF-β levels in supernatant did not increase [27]. This fits with the present study where we show that there is increased activation of TGF-β, rather than production after stimulation of macrophages with oxLDL. Using supernatants of oxLDL stimulated macrophages, strongly elevated levels of active TGF-β and, to a lesser extent, BMP were measured. Also *in vivo*, LDLr^−/−^ mice show an increased presence of TGF-β compared to WT mice, suggesting that this *in vitro* observation could very well reflect the *in vivo* mechanism. TGF-β was measured using a CAGA-Luc assay in which the CAGA promoter is coupled to a luciferase gene which becomes activated by active TGF-β via TGF-β receptor 1. TGF-β is released in an inactive form and can be activated by enzymes such as MMP2 and MMP9 [48]. These enzymes may be released either by activated synovial macrophages or by synovial fibroblasts in response to pro-inflammatory factors released by the macrophage. Recently, Ishikawa *et al*. demonstrated that apart from macrophages in rheumatoid arthritis fibroblast-like synoviocytes can also accumulate oxLDL via LOX-1, resulting in massive MMP production [49]. Studies are now in progress to define the factors involved in TGF-β activation. In addition to TGF-β activation, increased BMP activation was found after stimulation of macrophages with oxLDL. Enhanced BMP presence can also contribute to the observed bone formation, since previous studies showed that TGF-β is important for initiating osteophyte formation, whereas BMP are mainly involved at later stages in the process of endochondral ossification [50]. Also osteoarthritic chondrocytes have been shown to express LOX-1 and could, therefore, be affected by oxLDL directly [51]. Although our data support that (ox)LDL affects chondrocytes indirectly via synovial activation, it would be interesting to investigate how hypercholesterolemia could influence the chondrocytes directly and how this affects OA development.

We did not find osteophyte or enthesophyte formation in naïve knee joints of mice with an LDLr deficiency receiving a cholesterol-rich diet (data not shown). This would suggest that systemically enhanced LDL levels alone are not sufficient to induce joint pathology. Only when there are pro-inflammatory or damage associated stimuli present (that is, during OA or instability), may enhanced LDL levels result in increased osteophyte/enthesophyte formation.

## Conclusions

This study shows for the first time that increased LDL cholesterol levels in an OA milieu are able to enhance ectopic bone formation. Our experimental data points towards a potential mechanism in which uptake of oxLDL by synovial lining macrophages results in activation of TGF-β and to a lesser extent BMP. Further research is needed in order to elucidate what factors in particular are responsible for the enhanced levels of active TGF-β and BMP, since direct production does not seem to increase according to mRNA expression levels. Nevertheless, this mechanism provides a firm step forward to unraveling possible factors affecting etiopathology of OA and osteophyte formation in particular.

## Abbreviations

ApoB: Apolipoprotein B; BMP: Bone morphogenetic proteins; BRE-Luc: Bone morphogenetic protein responsive element luciferase reporter plasmid; BSA: Bovine serum albumin; CAGA-Luc: CAGA-box luciferase reporter plasmid; Ct: Threshold cycle; DAB: Diaminobenzidine; (D)MEM: (Dulbecco’s) Modified Eagle’s Medium; FCS: Fetal calf serum; GFP: Green fluorescent protein; HDL: High-density lipoprotein; H & E: hematoxylin and eosin; IL: Interleukin; LDL: Low-density lipoprotein; LDLr−/−: Low-density lipoprotein receptor deficient; LOX-1: Lectin-like oxidized low-density lipoprotein receptor-1; M-CSF: Macrophage colony stimulating factor; MMP: Metalloproteinase; OA: Osteoarthritis; oxLDL: Oxidized low-density lipoprotein; PBS: Phosphate-buffered saline; qPCR: Quantitative real-time polymerase chain reaction; TGF-β: Transforming growth factor-β; TNF-α: Tumor necrosis factor-alpha; WT: Wild type.

## Competing interests

The authors declare that they have no competing interests.

## Authors’ contributions

WdM, AB and PvL were involved in conception and design of the study, acquisition and analysis of data, and interpretation of data. MH and BW were involved in performing animal experiments and acquisition of histological data. PvdK, LJ and WvdB were involved in conception and design of the study. All authors have been involved in revising the manuscript. All authors read and approved the final manuscript.

## References

[B1] Felson DT Chaisson CE Understanding the relationship between body weight and osteoarthritis Baillieres Clin Rheumatol 1997 15 671 681 10.1016/S0950-3579(97)80003-9 9429730

[B2] Velasquez MT Katz JD Osteoarthritis: another component of metabolic syndrome? Metab Syndr Relat Disord 2010 15 295 305 10.1089/met.2009.0110 20367223

[B3] Hunter DJ Felson DT Osteoarthritis BMJ 2006 15 639 642 10.1136/bmj.332.7542.639 16543327 PMC1403209

[B4] Yusuf E Nelissen RG Ioan-Facsinay A Stojanovic-Susulic V DeGroot J van Osch G Middeldorp S Huizinga TW Kloppenburg M Association between weight or body mass index and hand osteoarthritis: a systematic review Ann Rheum Dis 2010 15 761 765 10.1136/ard.2008.106930 19487215

[B5] Katz JD Agrawal S Velasquez M Getting to the heart of the matter: osteoarthritis takes its place as part of the metabolic syndrome Curr Opin Rheumatol 2010 15 512 519 10.1097/BOR.0b013e32833bfb4b 20592604

[B6] Carman WJ Sowers M Hawthorne VM Weissfeld LA Obesity as a risk factor for osteoarthritis of the hand and wrist: a prospective study Am J Epidemiol 1994 15 119 129 8296779 10.1093/oxfordjournals.aje.a116974

[B7] Grotle M Hagen KB Natvig B Dahl FA Kvien TK Obesity and osteoarthritis in knee, hip and/or hand: an epidemiological study in the general population with 10 years follow-up BMC Musculoskelet Disord 2008 15 132 10.1186/1471-2474-9-132 18831740 PMC2573886

[B8] Koskinen J Magnussen CG Wurtz P Soininen P Kangas AJ Viikari JS Kahonen M Loo BM Jula A Ahotupa M Lehtimäki T Ala-Korpela M Juonala M Raitakari OT Apolipoprotein B, oxidized low-density lipoprotein, and LDL particle size in predicting the incidence of metabolic syndrome: the Cardiovascular risk in Young Finns study Eur J Prev Cardiol 2012 15 1296 1303 10.1177/1741826711425343 21960651

[B9] Mishra R Singh A Chandra V Negi MP Tripathy BC Prakash J Gupta V A comparative analysis of serological parameters and oxidative stress in osteoarthritis and rheumatoid arthritis Rheumatol Int 2012 15 2377 2382 10.1007/s00296-011-1964-1 21644045

[B10] Oliviero F Lo Nigro A Bernardi D Giunco S Baldo G Scanu A Sfriso P Ramonda R Plebani M Punzi L A comparative study of serum and synovial fluid lipoprotein levels in patients with various arthritides Clin Chim Acta 2012 15 303 307 10.1016/j.cca.2011.10.019 22037510

[B11] Badimon L Storey RF Vilahur G Update on lipids, inflammation and atherothrombosis Thromb Haemost 2011 15 S34 S42 10.1160/THS10-11-0717 21479344

[B12] Badimon L Vilahur G LDL-cholesterol versus HDL-cholesterol in the atherosclerotic plaque: inflammatory resolution versus thrombotic chaos Ann N Y Acad Sci 2012 15 18 32 10.1111/j.1749-6632.2012.06480.x 22548566

[B13] Morel DW Hessler JR Chisolm GM Low density lipoprotein cytotoxicity induced by free radical peroxidation of lipid J Lipid Res 1983 15 1070 1076 6415194

[B14] James MJ van Reyk D Rye KA Dean RT Cleland LG Barter PJ Jessup W Low density lipoprotein of synovial fluid in inflammatory joint disease is mildly oxidized Lipids 1998 15 1115 1121 10.1007/s11745-998-0313-8 9870907

[B15] Groeneweg M Kanters E Vergouwe MN Duerink H Kraal G Hofker MH de Winther MP Lipopolysaccharide-induced gene expression in murine macrophages is enhanced by prior exposure to oxLDL J Lipid Res 2006 15 2259 2267 10.1194/jlr.M600181-JLR200 16840796

[B16] van Tits LJ Stienstra R van Lent PL Netea MG Joosten LA Stalenhoef AF Oxidized LDL enhances pro-inflammatory responses of alternatively activated M2 macrophages: a crucial role for Kruppel-like factor 2 Atherosclerosis 2011 15 345 349 10.1016/j.atherosclerosis.2010.11.018 21167486

[B17] Jiang Y Wang M Huang K Zhang Z Shao N Zhang Y Wang W Wang S Oxidized low-density lipoprotein induces secretion of interleukin-1beta by macrophages via reactive oxygen species-dependent NLRP3 inflammasome activation Biochem Biophys Res Commun 2012 15 121 126 10.1016/j.bbrc.2012.07.011 22796220

[B18] Yoshida H Kondratenko N Green S Steinberg D Quehenberger O Identification of the lectin-like receptor for oxidized low-density lipoprotein in human macrophages and its potential role as a scavenger receptor Biochem J 1998 15 9 13 9693095 10.1042/bj3340009 PMC1219654

[B19] Murphy JE Vohra RS Dunn S Holloway ZG Monaco AP Homer-Vanniasinkam S Walker JH Ponnambalam S Oxidised LDL internalisation by the LOX-1 scavenger receptor is dependent on a novel cytoplasmic motif and is regulated by dynamin-2 J Cell Sci 2008 15 2136 2147 10.1242/jcs.020917 18544637

[B20] Bondeson J Blom AB Wainwright S Hughes C Caterson B van den Berg WB The role of synovial macrophages and macrophage-produced mediators in driving inflammatory and destructive responses in osteoarthritis Arthritis Rheum 2010 15 647 657 20187160 10.1002/art.27290

[B21] Bondeson J Wainwright SD Lauder S Amos N Hughes CE The role of synovial macrophages and macrophage-produced cytokines in driving aggrecanases, matrix metalloproteinases, and other destructive and inflammatory responses in osteoarthritis Arthritis Res Ther 2006 15 R187 10.1186/ar2099 17177994 PMC1794533

[B22] Blom AB van Lent PL Libregts S Holthuysen AE van der Kraan PM van Rooijen N van den Berg WB Crucial role of macrophages in matrix metalloproteinase-mediated cartilage destruction during experimental osteoarthritis: involvement of matrix metalloproteinase 3 Arthritis Rheum 2007 15 147 157 10.1002/art.22337 17195217

[B23] Rogers J Shepstone L Dieppe P Bone formers: osteophyte and enthesophyte formation are positively associated Ann Rheum Dis 1997 15 85 90 10.1136/ard.56.2.85 9068279 PMC1752321

[B24] van Beuningen HM Glansbeek HL van der Kraan PM van den Berg WB Differential effects of local application of BMP-2 or TGF-beta 1 on both articular cartilage composition and osteophyte formation Osteoarthritis Cartilage 1998 15 306 317 10.1053/joca.1998.0129 10197165

[B25] van Beuningen HM Glansbeek HL van der Kraan PM van den Berg WB Osteoarthritis-like changes in the murine knee joint resulting from intra-articular transforming growth factor-beta injections Osteoarthritis Cartilage 2000 15 25 33 10.1053/joca.1999.0267 10607496

[B26] Blom AB van Lent PL Holthuysen AE van der Kraan PM Roth J van Rooijen N van den Berg WB Synovial lining macrophages mediate osteophyte formation during experimental osteoarthritis Osteoarthritis Cartilage 2004 15 627 635 10.1016/j.joca.2004.03.003 15262242

[B27] van Lent PL Blom AB van der Kraan P Holthuysen AE Vitters E van Rooijen N Smeets RL Nabbe KC van den Berg WB Crucial role of synovial lining macrophages in the promotion of transforming growth factor beta-mediated osteophyte formation Arthritis Rheum 2004 15 103 111 10.1002/art.11422 14730606

[B28] Getz GS Reardon CA Animal models of atherosclerosis Arterioscler Thromb Vasc Biol 2012 15 1104 1115 10.1161/ATVBAHA.111.237693 22383700 PMC3331926

[B29] Ishibashi S Brown MS Goldstein JL Gerard RD Hammer RE Herz J Hypercholesterolemia in low density lipoprotein receptor knockout mice and its reversal by adenovirus-mediated gene delivery J Clin Invest 1993 15 883 893 10.1172/JCI116663 8349823 PMC294927

[B30] van der Kraan PM Vitters EL van Beuningen HM van de Putte LB van den Berg WB Degenerative knee joint lesions in mice after a single intra-articular collagenase injection. A new model of osteoarthritis J Exp Pathol 1990 15 19 31 PMC1998679 2155638

[B31] Pritzker KP Gay S Jimenez SA Ostergaard K Pelletier JP Revell PA Salter D van den Berg WB Osteoarthritis cartilage histopathology: grading and staging Osteoarthritis Cartilage 2006 15 13 29 10.1016/j.joca.2005.07.014 16242352

[B32] Glasson SS Chambers MG Van Den Berg WB Little CB The OARSI histopathology initiative - recommendations for histological assessments of osteoarthritis in the mouse Osteoarthritis Cartilage 2010 15 S17 S23 20864019 10.1016/j.joca.2010.05.025

[B33] van Tits LJ Demacker PN de Graaf J Hak-Lemmers HL Stalenhoef AF: alpha-tocopherol supplementation decreases production of superoxide and cytokines by leukocytes ex vivo in both normolipidemic and hypertriglyceridemic individuals Am J Clin Nutr 2000 15 458 464 10648258 10.1093/ajcn/71.2.458

[B34] Chomczynski P Sacchi N Single-step method of RNA isolation by acid guanidinium thiocyanate-phenol-chloroform extraction Anal Biochem 1987 15 156 159 2440339 10.1006/abio.1987.9999

[B35] van Lent PL Blom AB Schelbergen RF Sloetjes A Lafeber FP Lems WF Cats H Vogl T Roth J van den Berg WB Active involvement of alarmins S100A8 and S100A9 in the regulation of synovial activation and joint destruction during mouse and human osteoarthritis Arthritis Rheum 2012 15 1466 1476 10.1002/art.34315 22143922

[B36] Tan AL Grainger AJ Tanner SF Shelley DM Pease C Emery P McGonagle D High-resolution magnetic resonance imaging for the assessment of hand osteoarthritis Arthritis Rheum 2005 15 2355 2365 10.1002/art.21210 16052535

[B37] Chung SW Kang BY Kim SH Pak YK Cho D Trinchieri G Kim TS Oxidized low density lipoprotein inhibits interleukin-12 production in lipopolysaccharide-activated mouse macrophages via direct interactions between peroxisome proliferator-activated receptor-gamma and nuclear factor-kappa B J Biol Chem 2000 15 32681 32687 10934192 10.1074/jbc.M002577200

[B38] Mahley RW Innerarity TL Rall SC Jr Weisgraber KH Plasma lipoproteins: apolipoprotein structure and function J Lipid Res 1984 15 1277 1294 6099394

[B39] Goldstein JL Brown MS The LDL receptor Arterioscler Thromb Vasc Biol 2009 15 431 438 10.1161/ATVBAHA.108.179564 19299327 PMC2740366

[B40] Spady DK Meddings JB Dietschy JM Kinetic constants for receptor-dependent and receptor-independent low density lipoprotein transport in the tissues of the rat and hamster J Clin Invest 1986 15 1474 1481 10.1172/JCI112460 3700649 PMC424548

[B41] Mooney RA Sampson ER Lerea J Rosier RN Zuscik MJ High-fat diet accelerates progression of osteoarthritis after meniscal/ligamentous injury Arthritis Res Ther 2011 15 R198 10.1186/ar3529 22152451 PMC3334649

[B42] van Beuningen HM van der Kraan PM Arntz OJ van den Berg WB In vivo protection against interleukin-1-induced articular cartilage damage by transforming growth factor-beta 1: age-related differences Ann Rheum Dis 1994 15 593 600 10.1136/ard.53.9.593 7979598 PMC1005411

[B43] Yang H Mohamed AS Zhou SH Oxidized low density lipoprotein, stem cells, and atherosclerosis Lipids Health Dis 2012 15 85 10.1186/1476-511X-11-85 22747902 PMC3475066

[B44] Salvayre R Auge N Benoist H Negre-Salvayre A Oxidized low-density lipoprotein-induced apoptosis Biochim Biophys Acta 2002 15 213 221 10.1016/S1388-1981(02)00343-8 12531556

[B45] Chu L Hao H Luo M Huang Y Chen Z Lu T Zhao X Verfaillie CM Zweier JL Liu Z Ox-LDL modifies the behaviour of bone marrow stem cells and impairs their endothelial differentiation via inhibition of Akt phosphorylation J Cell Mol Med 2011 15 423 432 10.1111/j.1582-4934.2009.00948.x 19863696 PMC3822806

[B46] Lu T Parthasarathy S Hao H Luo M Ahmed S Zhu J Luo S Kuppusamy P Sen CK Verfaillie CM Tian J Liu Z Reactive oxygen species mediate oxidized low-density lipoprotein-induced inhibition of oct-4 expression and endothelial differentiation of bone marrow stem cells Antioxid Redox Signal 2010 15 1845 1856 10.1089/ars.2010.3156 20836655 PMC2971633

[B47] Tie G Yan J Yang Y Park BD Messina JA Raffai RL Nowicki PT Messina LM Oxidized low-density lipoprotein induces apoptosis in endothelial progenitor cells by inactivating the phosphoinositide 3-kinase/Akt pathway J Vasc Res 2010 15 519 530 10.1159/000313879 20431300 PMC2945270

[B48] Yu Q Stamenkovic I Cell surface-localized matrix metalloproteinase-9 proteolytically activates TGF-beta and promotes tumor invasion and angiogenesis Gene Dev 2000 15 163 176 10652271 PMC316345

[B49] Ishikawa M Ito H Akiyoshi M Kume N Yoshitomi H Mitsuoka H Tanida S Murata K Shibuya H Kasahara T Kakino A Fujita Y Sawamura T Yasuda T Nakamura T Lectin-like oxidized low-density lipoprotein receptor 1 signal is a potent biomarker and therapeutic target for human rheumatoid arthritis Arthritis Rheum 2012 15 1024 1034 10.1002/art.33452 22076918

[B50] Blaney Davidson EN Vitters EL van Beuningen HM van de Loo FA van den Berg WB van der Kraan PM Resemblance of osteophytes in experimental osteoarthritis to transforming growth factor beta-induced osteophytes: limited role of bone morphogenetic protein in early osteoarthritic osteophyte formation Arthritis Rheum 2007 15 4065 4073 10.1002/art.23034 18050218

[B51] Simopoulou T Malizos KN Tsezou A Lectin-like oxidized low density lipoprotein receptor 1 (LOX-1) expression in human articular chondrocytes Clin Exp Rheumatol 2007 15 605 612 17888218

